# Supplement C18:1 in Culture Medium to Improve Survival Rate and Fermentation Activity of Lyophilization of *Lacticaseibacillus paracasei* L9

**DOI:** 10.4014/jmb.2505.05007

**Published:** 2025-08-28

**Authors:** Weina Miao, Li Lin, Yanling Hao, Zhengyuan Zhai, Ran Wang, Jingjing Liang, Ming Zhang, Yue Sang, Hongliang Li, Lianzhong Ai, Liang Zhao

**Affiliations:** 1 College of Food Science and Nutritional Engineering, China Agricultural University, Beijing 100083, P.R. China; 2 Key Laboratory of Functional Dairy, Department of Nutrition and Health, China Agricultural University, Beijing 100193, P.R. China; 3 Research Center for Probiotics, China Agricultural University, Beijing 101299, P.R. China; 4 School of Food and Health, Beijing Technology and Business University, Beijing 100048, P.R. China; 5 Inner Mongolia Mengniu Dairy (Group) Co., Ltd, Hohhot 011517, P.R. China; 6 Shanghai Engineering Research Center of Food Microbiology, School of Health Science and Engineering, University of Shanghai for Science and Technology, Shanghai 200093, P.R. China

**Keywords:** Lactic acid bacteria, freeze-drying, fermentation activity, fatty acid, cell membrane

## Abstract

This study explored the impact of C18:1 fatty acid supplementation in culture medium on the survival rate, fermentation activity, and membrane stability of *Lacticaseibacillus paracasei* L9 during freeze-drying. Cells cultured in medium supplemented with C18:1 (0.2 mg/ml) exhibited a higher survival rate (95.38%) after freeze-drying compared to the control (83.56%). The fermentation activity of the freeze-dried powder was significantly enhanced, with improvements in viable count (Δ viable counts: 1.79 Log CFU/ml), pH reduction (Δ pH: 2.03), and titratable acidity (Δ titratable acidity: 99.4 °T) in skim milk. Supplementation with C18:1 increased the activity of key enzymes, such as *β*-galactosidase (13.25 U/ml) and lactate dehydrogenase (2.16 U), while reducing cell membrane permeability. The addition of C18:1 also modulated the expression of membrane synthesis-related genes, including *plsX* and *YfhO*, and altered the membrane fatty acid composition by increasing the proportion of unsaturated fatty acids (UFA). Specifically, the proportion of UFA in the membrane increased from 53.36% in the freeze-dried control to 63.28% in the C18:1-supplemented group. These results indicate that C18:1 supplementation improves cell membrane stability and fermentation activity by modulating gene expression and membrane fatty acid composition, thereby enhancing the freeze-drying tolerance of *L. paracasei* L9.

## Introduction

Lactic acid bacteria (LAB) are the most prevalent starter culture, essential for the production of various fermented foods such as yogurt, cheese, pickles, and beverages like kefir and kumis, while also providing multiple health-promoting functions [1]. For industrial applications, it is crucial to maintain LAB viability, acidification capacity, and enzymatic activity over storage, as these properties underpin their fermentative and probiotic functions. Freeze-drying is a commonly employed preservation method for LAB starter cultures due to its advantages in genetic stability, shelf life, and rehydration efficiency [2]. Compared to other drying techniques such as spray-drying or fluidized bed drying, freeze-drying typically yields higher survival rates [3]. However, the process imposes substantial stress, including low temperature and dehydration, which can damage cell membranes, denature proteins and enzymes, and reduce membrane integrity, fluidity, and permeability—thereby compromising both viability and, more critically, fermentative performance [4, 5].

While the survival rate of freeze-dried LAB powders is commonly used as the primary indicator of starter quality, fermentation activity is equally critical for evaluating their functional performance. Freeze-drying can cause severe damage leading directly to cell death, leading to a reduction in viability and increasing the number of cultivation cycles needed to obtain high-viability fermenters—thus raising production costs and becoming a major focus of research. In fact, damages that do not immediately result in death may still compromise fermentative performance, which cannot be reflected by viability measurements alone. Maite Gagneten *et al*. [6] found that the specific acidifying activity of *L. plantarum* and *L. bulgaricus* increased after freeze-drying. This indicated that with the same viable cell count, the fermentation activity of a single freeze-dried cell is reduced, as a result of the loss of culturability and decrease of the vitality. Moreover, Chen *et al*. [7] showed that the fermentation performance of freeze-dried *L. rhamnosus* and *L. fermentum* was influenced by the effects of pre-freezing temperatures on the activities of their lactate dehydrogenase (LDH) and ATPase. These often-overlooked impairments in fermentation activity may lead to prolonged fermentation time and increased starter dosage in order to meet production standards (*e.g.*, final pH 4.2–4.35), which directly impact industrial cost and efficiency. Therefore, preserving the fermentative activity of freeze-dried LAB is not only biologically relevant, but also critical to industrial performance. However, it has received comparatively less attention than viability in existing research.

Freeze-drying primarily affects the fermentation activity of strains through causing cell membrane damage, which plays a central role in maintaining enzymatic activity. As the structural barrier between the intracellular environment and external stress, the membrane is particularly susceptible to damage during the freeze-drying process [8]. More importantly, it serves as the key interface for fermentation-related metabolic processes, including the transport of lactose and its subsequent hydrolysis by intracellular enzymes. Qiao *et al*. [9] observed by transmission electron microscopy that the cell membrane of *S. thermophilus* partially disappeared after freezing and the fluidity decreased. Such decreases in membrane fluidity can lead to conformational changes and dysfunction in membrane-bound proteins. Enzymes such as lactose permease and Na^+^/K^+^-ATPase [10], which are integral to the membrane and essential for substrate uptake and energy transduction, become dysfunctional under these conditions. Also, Li *et al*. [11] reported increased membrane permeability and reduced integrity in LAB following freeze-drying, which led to loss of proton motive force [12] and reduced activities of fermentation-related enzymes. Collectively, these findings demonstrate that the fermentative capacity of LAB is closely dependent on membrane stability (specifically its integrity, fluidity, and selective permeability), all of which are vulnerable to freeze-drying stress.

Given the critical role of membrane stability in maintaining fermentative function, the development of protective agents to mitigate freeze-drying damage has attracted significant attention. For instance, trehalose [13] and sorbitol [14] can help improve membrane fluidity and stability by forming hydrogen bonds or increasing the glass transition temperature. However, these additives primarily offer passive protection during the freeze-drying process and their efficacy and options is often limited and strain-specific. The composition of cell membrane fatty acids has great impact on cell membrane fluidity and stability. The alterations of membrane fatty acids in saturate, carbon chain length, cis/trans isomerization, or cyclopropanation influenced the fluidity and freeze-drying stability of the cell membrane [15]. But during the freeze-drying process, bacterial cells are exposed to extreme stresses and are thus unable to modify their membrane properties in order to limit damages [16]. In contrast, the culture process of a strain is a crucial phase in cell membrane formation [17]. Optimizing culture conditions not only enhances the stress resistance of strains but also offers broader universality and more comprehensive protection of functional characteristics [10]. This can be achieved by adding functional nutrients and factors, as well as optimizing fermentation parameters (such as pH and temperature). For instance, studies [18] have shown that the presence of K^+^ or optimizing culture medium by adding uracil [19] can contribute to cell membrane formation [20] and enhance the survival rate; and adjusting the fermentation pH (*e.g.*, 6.8 and 7.4) [21] can shift membrane fatty acid composition—such as increasing the ratio of unsaturated to saturated fatty acids and cyclopropane fatty acid content—thereby enhancing membrane mobility. Despite these advances, most studies have focused on improving survival, while relatively little attention has been paid to enhancing the fermentation activity of freeze-dried LAB. Moreover, the mechanistic connection between membrane stability and fermentative performance remains poorly defined. These gaps highlight the need for strategies that proactively enhance both viability and functional activity by targeting membrane properties at the cultivation stage.

*Lacticaseibacillus paracasei* L9, a probiotic strain derived from healthy human feces, has been demonstrated to possess beneficial properties, such as regulating immune imbalances in food allergies, modulating human intestinal function, and elevating the levels of short-chain fatty acids in the gut [22]. These attributes, coupled with its robust fermentation performance, underscore as a fermentation strain in fermented milk beverage. This study investigates the influence of different fatty acids supplemented in the culture medium on the fermentation activity of freeze-dried *L. paracasei* L9, with a particular focus on the role of membrane stability and enzymatic activity. By targeting membrane composition during the cultivation phase, this approach aims to enhance both viability and functional performance. The findings are expected to support the development of freeze-dried formulations of *L. paracasei* L9 with improved fermentative capacity.

## Materials and Methods

### Bacterial Strain and Inoculum Preparation

Stock culture of *L. paracasei* L9 (CGMCC No. 9800) was stored in glycerol at −80°C. The 200 μl of stock culture were transferred to 10 ml of de Man, Rogosa and Sharpe (MRS, Land Bridge, China) broth and cultured at 37°C for 12 h as the inoculum.

### Fatty Acid Supplemental Media and Growth Conditions

The strain was cultivated in MRS-Lac broth (replacing glucose in MRS with equal quantity lactose) supplemented with varying fatty acids: oleic acid (C18:1), palmitoleic acid (C16:1), and palmitic acid (C16:0). The fatty acids were diluted in ethanol as liquid storage and sterilized with 0.22 μm filter. Then 0, 50, 100, 150, 200 μl of the corresponding fatty acid ethanol solutions were added in 10 ml sterilized MRS-Lac broth respectively, to prepare different concentrations of fatty acid supplemental media. An equivalent volume of ethanol was also added to the medium, designated as the Solvent group. The ordinary MRS-Lac medium without fatty acids supplemented or ethanol was served as the control (referred as Liquid Culture group).

The L9 inoculum was inoculated in MRS-Lac medium with/without fatty acid supplemental media or solvent at a density of 5×10^7^ colony-forming units (CFU)/ml and cultured at 37°C. Then cells of 20 ml of broth were harvested by centrifugation (4°C, 7,000 ×*g* for 5 min) after cultured 12 h corresponding to the end of exponential phase. The pelleted cells were washed twice with saline solution (0.85% NaCl), and the resulting bacterial precipitate was processed for the preparation of lyophilized powder.

### Preparation of Lyophilized Powder

The process was based on the method described by Pei *et al*. [23] with some modification. The bacterial precipitate was thoroughly mixed with protectant at an equal weight ratio (1:1, w/w). The protectant solution was composed of 15% (w/v) sucrose, 5% (w/v) trehalose, 2% (w/v) sodium glutamate, and 8% (w/v) skim milk with distilled water constituting the remaining 70% (w/v) of the solution. The fat content of skim milk (D8340) purchased from Solarbio was less than 1.0%, and it was reduced to 0.05% after mixed in protectant, with the residual fat primarily consisting of short-chain fatty acids [24]. Subsequently, the mixtures were subjected to a freeze-drying pre-freezing step by being placed at -80°C for 4 h.

The lyophilization was conducted in a vacuum freeze-dryer (FreeZone, LABCONCO, USA), with the cold trap set at –50°C and the chamber vacuum degree maintained at 5 Pa for a duration of 48 h. Bacterial suspensions from the Liquid Culture and C18:1-supplemented groups were processed under identical conditions, and the resulting powders were designated as FD and FD-C18:1, respectively. Freshly lyophilized powders were used immediately for subsequent experiments, or temporarily stored at –20°C if short-term waiting was necessary. For experiments requiring repeated trials, portions of the powder were sealed under strict anaerobic conditions and stored at –80°C for no more than two weeks before use.

For rehydration, each tube of lyophilized cells was rehydrated to their pre-lyophilization volume (20 ml) using saline solution (0.85% NaCl) and thoroughly homogenized using a vortex mixer (MS2, IKA Works, Germany) for 5 min at 25°C, in preparation for subsequent analyses.

### Survival Rate of L9 after Lyophilization

The survival rate of the bacteria was assessed through viable counts obtained before and after the freeze-drying processes by the plate counting [24]. The rehydration solutions of Liquid Culture, FD and FD-C 18:1 group were serially diluted in sterile saline (0.85% NaCl). Aliquots (1 ml) of each dilution were poured into MRS agar plates in triplicate. The inoculated plates were cultured at 37°C for 48 h. The colonies were enumerated and reported as colony-forming units per milliliter (Log CFU/ml). The bacterial survival rate was then calculated using the following formula:

$$\text{Survival rate}\left(\%\right)=\frac{N}{N_{0}}\times 100\%$$

Where, N_0_ represents the log of colony forming units before freeze-drying; N represents the log of colony forming units after freeze-drying; All experiments were carried out in triplicate.

### Determination of Fermentation Activity of Freeze-Dried Powder

For all groups—Liquid Culture, Freezing (Control, C18:1), and FD (Control, C18:1)—bacterial cells were collected by centrifugation (4 000 ×*g*, 10 min, 4°C) and washed twice with sterile saline (0.85% NaCl). The washed cells were then resuspended and adjusted to an equivalent initial viable count (5 × 10^7^ CFU /ml), and inoculated into the skim milk (12% w/w) at the same volume (2%, v/v) for fermentation at 37°C for 24 h. And the pH, viable counts, and titratable acidity were determined before and after fermentation. Then the changes of pH (decrements), viable counts and titratable acidity (increments) were calculated to characterize fermentation activity. To measure the titratable acidity, 10 ml of the sample was diluted with 20 ml of distilled water and thoroughly mixed. The mixture was titrated with 0.1 M NaOH to a stable pH of 8.30 ± 0.02 for at least 30 sec [25]. The titratable acidity of the sample was calculated using the following equation:

$$\text{Titratable acidity }\left({}^{\circ}T\right)=\frac{c\times v_{1}\times 1000}{v_{0}}mmol/L$$

Where, *c* = the concentration of the NaOH standard solution (0.1 mol/l), *v_1_* = the volume of NaOH consumed (ml), *v_0_* = the volume of the test sample (10 ml).

Each measurement was performed in triplicate, and the mean value was determined.

### Analyses of Fatty Acid Composition

The relative fatty acid composition of L9 was determined before and after freeze-drying by gas chromatography using the method described by Cheng [14] with some modifications. Lipids were extracted as described by Bligh and Dyer [26] with some modification. Before collecting cell pellets, 10 ml aliquots of both Liquid Culture and rehydration solutions of freeze-dried powder (FD and FD-C18:1) were washed in PBS twice at 8,000 × g for 10 min to remove residual lyoprotectant or medium components and avoid interference from these components.

The bacterial pellet (100 mg) was transferred into a 2 ml grinding tube containing 0.5 mm steel beads. Add 1 ml of a chloroform: methanol mixture (1:1, v/v), place the tube into a freeze grinder (50 Hz) for 3 min and centrifugate it. The supernatant was drawn into a 1.5 ml EP tube, and dried by nitrogen. 0.5 ml methylation reagent, namely sodium hydroxide methanol solution (0.5 mol/l) was added, vortexed for 30 sec and water bath at 60°C for 0.5 h. After cooled, 0.5 ml of n-hexane was added, vortexed for 30 sec, centrifuged for 10 min (4°C, 7,000 × g), and the upper layer was taken and stored at −80°C until analysis. The extracted supernatant samples were taken into the sample injection vial and detected using an 8890B-5977B GC–MS system (Agilent Technologies, USA) equipped with a CPSIL88 Capillary column (100 m, 0.25 mm, 0.25 μm, Agilent J&W Scientific, USA).

The concentration of individual fatty acids was calculated by peak area of the sample analyte and corresponding linear equation. And we calculated the following parameters: the relative content of UFAs, MUFAs and very-long-chain fatty acids (VLCFAs, ≥18 carbons) to characterize the fatty acid composition of samples.

### Enzyme Activity Assay

The crude cell-free extract was prepared from the samples of Liquid Culture, FD, and FD-C18:1. Cells were harvested by centrifugation (4,000 × g, 4°C, 15 min), washed once, and resuspended in PBS (pH 7.2). The cell suspensions (1 ml) were disrupted using a MiniBeadbeater-16 (Biospec, USA) in a 4°C ice bath at a speed of 5 m/s for two cycles (25 sec each, with a 10 sec pause), using 0.1 mm glass beads. Cell debris was removed by centrifugation (10,000 ×*g*, 10 min, 4°C), and the resulting cell-free extract was used for enzymatic assays [25]. The *β*-galactosidase (*β*-GAL) activity assay was performed as described by Liu *et al*. [27] 0.1 ml of the crude extract from each sample was used in the assay. *β*-GAL activity was expressed as the standard unit of U/ml, where one unit (U) corresponds to the formation of 1 μmol of ONP per minute. The lactate dehydrogenase (LDH) activity assay was conducted following the method of Zhu *et al*. [28]. For this assay, 10 μl of enzyme solution from each sample was used, and absorbance at 340 nm (A_340_) was continuously measured every 0.5 min for a total of 3 min. The control group contained no enzyme solution. One unit (U) of LDH activity was defined as each 1 ml enzyme solution (obtained from 1 ml bacteria solution in each sample) decreased A_340_ in unit time, namely U = ΔA_340_/min. All enzyme assays were performed in triplicate.

### Cytoplasmic Membrane Permeability

Proton motive force (PMF) of cell membrane was used to indicate cytoplasmic membrane permeability by measuring changes of fluorescence intensity after labeling with fluorescent probes. Aliquots of 1 ml bacteria taken from Liquid Culture, FD and FD-C18:1 were centrifuged at 4°C (10,000 × g, 5 min), removed supernatant and resuspended in 10 mmol/l glucose solution to 1 ml for detection. Cytoplasmic membrane permeability was determined according to the method of Liu *et al*. [29] with some modifications. The cell suspension was incubated with 0.4 μM (final concentration) fluorescent dye (DiSC3-5, Shanghai Maokang Biotechnology Co., Ltd., China) and the fluorescence intensity was scanned every 30 sec to determine the transmembrane electrical potential (Δψ). The transmembrane pH gradient (ΔpH) assay. 40 μl cell suspension from each sample and 1 μl fluorescent pH indicator BCECF AM (5 mg/ml, S1006, Beyotime Biotechnology, China) was used in this assay. The precipitates treated with concentrated HCl were washed with PBS once, centrifuged and finally suspended in 400 μl PBS. The fluorescence intensity was measured by a multifunctional microplate reader (Infinite 200 PRO, Tecan, Switzerland).

### Quantitative Real-Time PCR (qPCR) Analysis of Gene Expression

RNA was extracted from bacteria samples used the phenol-chloroform extraction method as previously described by Sun [30], and the concentration and purity of the extracted RNA were evaluated using a multifunctional microplate reader (Infinite M200Pro, Tecan). Subsequently, the All-In-One 5X RT Master Mix (Applied Biological Materials, Canada) was used to synthesis single-strand cDNA. Three technical replicates were used and for each reaction, the qPCR reaction mixture consisted of 10 μl of SYBR GreenI Real-time PCR master mixes (Takara, China), 0.5 μl of each forward and reverse primer (Table S1), 4 μl of nuclease-free water, and 5 μl of cDNA. The Lightcycler 96 instrument (Roche, USA) performed a quantitative polymerase chain reaction (qPCR). The comparative Ct technique (2^−ΔΔCT^) was used to quantify fold changes in gene expression between Liquid Culture, FD and FD-C18:1 group, and the samples were normalized to 16S rRNA expression. Gene expression levels were normalized to FD group, which served as the control with a value of 1.

### Statistical Analyses

Statistical analyses were performed using SPSS (SPSS Inc., USA, V 26.0.0). Data were expressed as mean ± standard error or standard deviation, using *t*-test or one-way analysis of variance ( ANOVA ) and Tukey test to compare all study groups and analysis values of *p* < 0.05 were considered as significant in Pearson correlation.

## Results

### Effect of Freeze-Drying on Growth and Fermentation Activity of *L. paracasei* L9

**Fig. 1:**
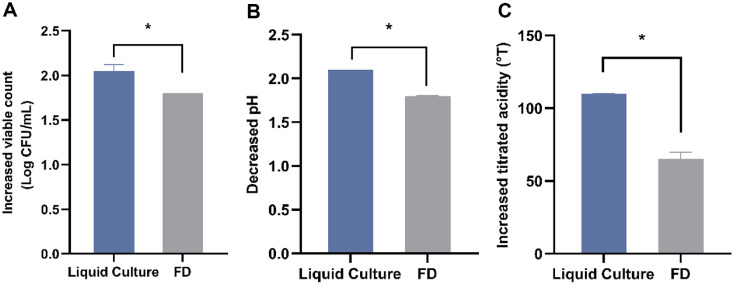
Fermentation activity of *Lacticaseibacillus paracasei* L9 before and after freeze-drying. Differences of viable count (**A**), pH (**B**) and titrated acidity (**C**) after 24 h fermentation. *Denotes statistical difference (*p* < 0.05), based on the *t*-tests. Error bars represent standard deviation obtained using independent triplicates.

The survival rate of L9 after freeze-dried was 83.5% ±1.03%. The fermentation activity of *L. paracasei* L9 (Lipid culture or FD) in skim milk was indicated by Δ viable count (A), Δ pH (B), and Δ titrated acidity (C) (Fig. 1).

Under the same initial viable counts, the fermentation activity of freeze-dried powder in FD is significantly lower than that of fresh bacterial liquid in Liquid Culture. FD demonstrated a significantly (*p* < 0.05) decrease of 0.25 (Log CFU/ml) in viable count, 0.3 units in pH and 45 °T in titrated acidity compared to control group Liquid Culture, after 24 h fermentation. Notably, the titrated acidity of FD decreased by 41%, reflecting a huge loss. It indicates that freeze-drying markedly reduced the fermentation activity of *L. paracasei* L9, especially the acid producing capacity (Fig. 1).

### Effect of Freeze-Drying on Membrane Fatty Acid Composition of *L. paracasei* L9

The percentages of membrane fatty acids in cells from Liquid Culture and FD were measured. A total of 10 fatty acids were identified and presented in Table 1.

As shown in Table 1, before and after freeze-drying, the distribution of fatty acids in the L9 cell membrane is similar, with the main components being the saturated fatty acids hexadecanoic acid (C16:0) and octadecanoic acid (C18:0), as well as the unsaturated fatty acid octadecenoic acid (C18:1). And the relative proportions of each fatty acid differed.

We found freeze-drying significantly altered specific membrane fatty acid compositions. Five fatty acids exhibited significant changed (*p* < 0.05). The levels of C18:1 and C18:2 decreased after freeze-drying, while only C16:1, C18:0 and C16:0 increased. It became clear that C18:1 and C16:0 primarily contributes to the downward trend in UFAs and MUFAs content and ratio. Additionally, C16:1 is the only unsaturated fatty acid that has increased. Based on the results, three increased fatty acids increased after freeze-drying—C16:0, C16:1, and C18:1—were selected to supple for further investigation.

### Effect of Fatty Acids Supplementation on the Gowth and Survival Rate of *L. paracasei* L9

**Fig. 2:**
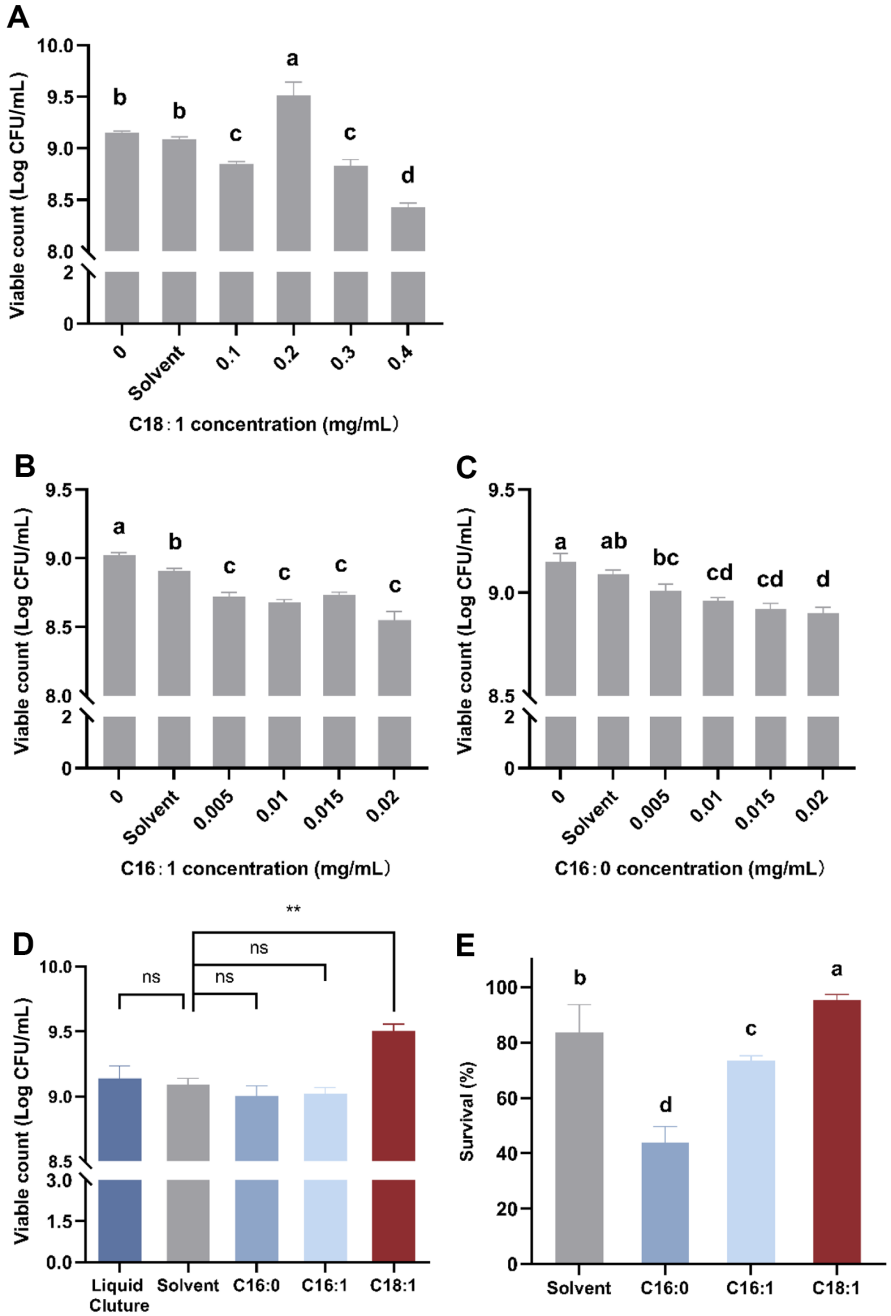
Viable count and freeze-drying survival rate of *Lacticaseibacillus paracasei* L9 grown with C18:1, C16:1, or C16:0 supplemented. In Fig. 2D and 2E, the supplement of fatty acids was C16:0 (0.005 mg/ml), C16:1(0.005 mg/ml), or C18:1(0.2 mg/ml). The solvent group is added with anhydrous ethanol as control. ^a-c^Different letters indicate significant differences (*p* < 0.05), based on the Tukey test. *Denotes significant differences (*p* < 0.05), **indicates very significant (*p* < 0.01), and “ns” indicates no significant difference, based on the one-way ANOVA. Error bars represent standard error obtained using independent triplicates.

To determine the effects of aforementioned three fatty acids on the growth of L9, gradient experiments were conducted to determine the optimal added concentration. The viable count of L9 grown with supplemented C18:1 (A), C16:1 (B), or C16:0 (C) was determined (Fig. 2).

The results demonstrated that C18:1 can promote the growth of L9 within a specific concentration range. At a concentration of 0.2 mg/ml, the promotion was maximized. However, both C16:0 and C16:1 inhibited the growth of L9, even at very low concentrations. In order to minimize the inhibitory effect of fatty acid supplementation on the growth of L9, suitable suppled concentrations were determined to be 0.005 mg/ml (C16:0), 0.005 mg/ml (C16:1), and 0.2 mg/ml (C18:1). The results in Fig. 2D demonstrated a consistent growth trend, that 0.2 mg/ml of C18:1 significantly increased (*p* < 0.05) the viable count of bacterial growth to 9.50 (Log CFU/ml), compared to the Liquid Culture and Solvent groups, which had viable counts of only 9.14 and 9.09 (Log CFU/ml), respectively. The C16:1 and C16:0 did not affect viable count in growth compared with control. The survival rate of C18:1 after freeze-drying was 95.38%, significantly (*p* < 0.05) higher than Solvent (83.56%), indicating that C18:1 can protect bacteria under freeze-drying stress, improving their survival.

### Effects of C18:1 Supplemented on the Fermentation and Enzyme Activity of Freeze-Dried *L. paracasei* L9

**Fig. 3:**
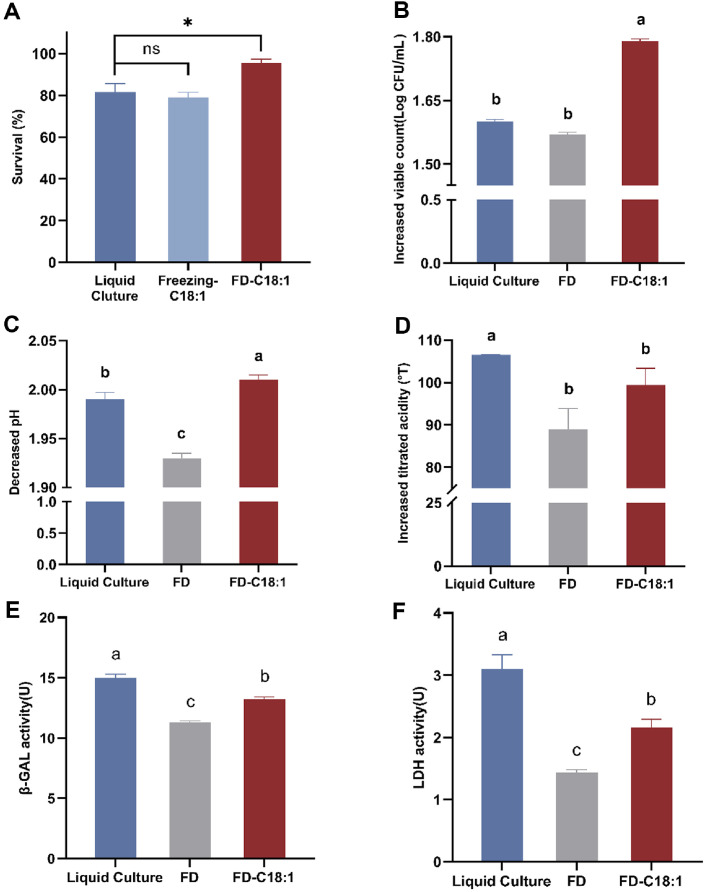
Survival (A) and fermentation activity of *Lacticaseibacillus paracasei* L9 grown with C18:1 supplemented: differences of viable count (B), pH (C) and titrated acidity (D) before and after 24 h fermentation; intracellular *β*-GAL (E) and LDH (F) activity. FD-indicates groups with freeze-drying treatment. ^a-c^Different letters indicate significant differences (*p* < 0.05) between freeze-dried and Liquid Culture of L9, based on the Tukey test. Error bars represent standard error obtained using independent triplicates.

Both freezing (identically pre-freezing at -80°C but not processed freeze-drying) and freeze-dried samples were assessed in Fig. 3. In terms of fermentation activity, C18:1 exerted a stronger protective effect. In Fig. 3B-3D, the Freezing-C18:1 had a higher value of Δ viable counts, Δ pH, and Δ titratable acidity than control, especially with a particularly significant improvement in titratable acidity. It implied C18:1 could improve the fermentation activity of frozen L9 in skim milk to a certain extent. Freeze-dried *L. paracasei* L9 grown with C18:1 supplementation (FD-C18:1) also exhibited higher fermentation activity compared to FD-Control, despite having the same initial viable count before fermentation. The Δ viable counts, Δ pH, and Δ titratable acidity for the freeze-dried samples were substantially improved from 1.63 to 1.79 (Log CFU/ml), 1.93 to 2.03 and 89 to 99.4°T. The data revealed that supplementation with C18:1 also significantly enhanced fermentation activity of freeze-dried *L. paracasei* L9 in skim milk

Notably, compared with Control, C18:1 enhanced the viable count and reduced the pH in post-fermentation of both Freezing and Freeze-dried, while it could not fully restore the decrease in titratable acidity caused by freeze-drying compared to the Liquid Culture. FD-C18:1 was higher than Liquid Culture in Δ viable counts and Δ pH but not Δ titratable acidity. Overall, it indicated that although C 18:1 could improve the fermentation activity of freeze-dried L9, the protect effect of C18:1 could not reverse the impact of freeze-dried on the ferment ability of L9.

Since fermentation activity is related to the activity of fermentation-related enzymes, the intracellular activity of *β*-glucosidase (Fig. 3E) and lactate dehydrogenase (Fig. 3F) in freeze-dried *L. paracasei* L9 grown with C18:1 supplementation was analyzed. With the addition of C18:1, the enzyme activities of L9 were better maintained compared to FD. *β*-GAL activity increased from 11.31 U (FD) to 13.25 U (FD-C18:1), and LDH activity increased from 1.43 U to 2.16 U. The results indicated that freeze-dried *L. paracasei* L9 supplemented with C18:1 exhibited a stronger ability to decompose lactose and produce lactic acid when compared with FD. However, the addition of C18:1 could not restore the damage to enzymatic activity caused by freeze-drying to the level Liquid Culture.

### Effect of C18:1 Supplemented on the Cell Membrane Stability of Freeze-Dried *L. paracasei* L9 Powder

**Fig. 4:**
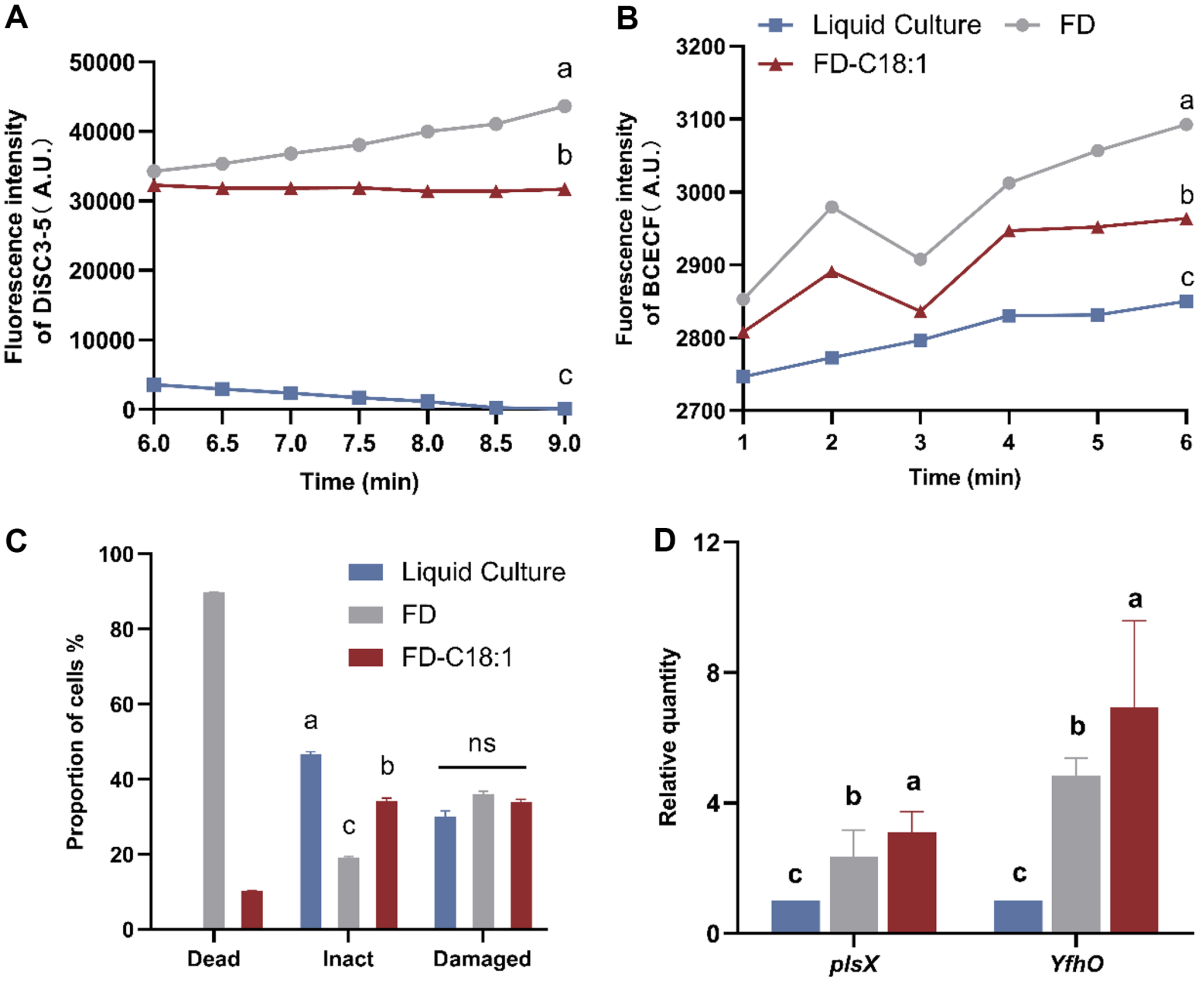
Effect of C18:1 supplemented on the cell membrane permeability of freeze-dried *Lacticaseibacillus paracasei* L9: ΔpH (A) and Δψ (B) of membrane; Cell membrane damage of different subgroups (C) Relative quantification of membrane-related genes (D). FD-indicates groups with freeze-drying treatment and the percentage of dead cell of Culture in Fig. 4D is too low to display. ^a-c^Different letters indicate significant differences (*p* < 0.05) between L9 with different treatments, “ns” indicates no significant difference, based on the Tukey test. Error bars represent standard error obtained using independent triplicates.

As shown in Fig. 4A, the Liquid Culture group exhibited the lowest and even continuously decreasing fluorescence intensity, suggesting that while maintaining the membrane potential, the phospholipid bilayer continued to bind with the fluorescent dye leading to dye quenching. The fluorescence intensity in FD group was higher than Liquid Culture and showed a continuous upward trend over the detection period, indicating freeze-dried L9 exhibited cell membrane damage with increased membrane permeability. This caused DiSC3-5 was released into the solution leading to an increase in fluorescence intensity. With the supplementation of C18:1, the fluorescence intensity of FD-C18:1 lower than that of FD and the trend remained stable, indicating that both groups exhibited increased fluorescence intensity due to the release of DiSC3-5 into the solution, while the addition of C18:1 resulted in a lower degree of cell membrane damage and a relatively stable membrane potential. FD-C18:1 group exhibited lower fluorescence than FD but higher than Liquid Culture (Fig. 4B), indicating that L9 grown with C18:1 supplied ameliorated the increased cell membrane permeability caused by freeze-drying, reduced the dissipation of the transmembrane potential (ΔpH), but did not restore the cells to their fresh state.

We utilized flow cytometry to further assess cell membrane damage in freeze-dried L9 bacteria at the subgroup level (Fig. S1). Additionally, we quantified the impact of C18:1 supplementation on L9 freeze-dried powder cells by enumerating dead, damaged, and intact bacteria in each group, as shown in Fig. 4C. The proportion of dead bacteria in freeze-dried powder cultured with C18:1 (FD-C18:1) was 30.10%, which was significantly lower than that of the control (46.68%) (*p* < 0.05). Conversely, the proportion of intact bacteria was 33.98%, which was significantly higher than that of FD (19.10%) (*p* < 0.05). And there was no significant difference in the proportion of damaged bacteria between the two groups. These findings indicated that the addition of C18:1 during the culture process can reduce cell membrane damage and death of L9 after freeze-drying, thereby improving the fermentation activity of the freeze-dried powder.

In order to further explore the effect of C18:1 on cell membrane freeze-drying damage, the expression of membrane synthesis-related genes was detected (Fig. 4D). Compared to the control (FD), the expression of the phosphate acyl-transferase *plsX* (LPL9_RS08660) and the bacterial membrane protein *YfhO* (LPL9_RS05860) was significantly elevated in FD-C18:1 (*p* < 0.05). This suggested that C18:1 supplementation enhanced the synthesis of cell membrane lipids and proteins. Collectively, these findings indicated that supplementation with C18:1 can mitigate membrane permeability and reduce cellular damage and mortality associated with freeze-drying in L9 cells, potentially through the enhancement of cell membrane synthesis.

### Effect of C18:1 Supplemented on Membrane Fatty Acid Composition and Related Gene Expression of Freeze-Dried *L. paracasei* L9 Powder

**Fig. 5:**
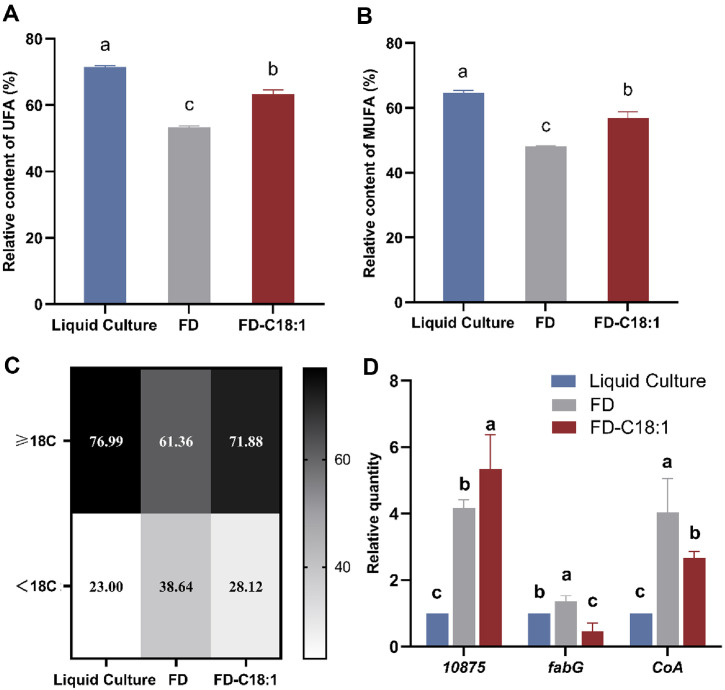
Membrane fatty acid composition and Relative quantification of fatty-acid-related genes of *Lacticaseibacillus paracasei* L9 before and after freeze-drying (FD). (**A**) Total unsaturated fatty acid; (**B**) monounsaturated fatty acid; (**C**) very-long-chain fatty acids (≥18C) and Medium- and short-chain fatty acids (MCFAs/SCFAs, <18 carbons); (**D**) relative expression of genes. FD- indicates groups with freeze-drying treatment. UFA: unsaturated fatty acid; MUFA: monounsaturated fatty acid; ≥18C: very-long-chain fatty acids (≥18C); <18C: Medium- and short-chain fatty acids (MCFAs/SCFAs, <18 carbons). ^a-c^Different letters indicate significant differences (*p* < 0.05) between L9 with different treatments, based on the Tukey test. Error bars represent standard error obtained using independent triplicates.

The membrane fatty acid composition of freeze-dried L9 with C18:1 was further analyzed. A total of twenty-two different fatty acids were identified and presented in Table S2. Significant differences in the proportions of membrane fatty acids were observed. To illustrate these changes more clearly, the contents of total unsaturated fatty acids (UFAs) (A), monounsaturated fatty acids (MUFAs) (B), and very-long-chain fatty acids (VLCFAs, ≥18 carbons) (C) on the membrane with C18:1 supplementation were quantified in Fig. 5A-5C.

The UFAs proportion of FD significantly decreased (*p* < 0.05) from 71.52% to 53.36% compared to Liquid Culture, especially the content of MUFAs (from 64.75% to 48.09%), indicating freeze-drying process decreased the content of UFAs on membrane and wherein MUFAs were the most significantly depleted. C18:1 supplementation significantly (*p* < 0.05) increased the proportion of UFA in FD-C18:1 to 63.28% and the proportion of MUFAs up to 56.83% compared to FD. Whereas the proportion of UFAs in the membrane still could not be restored to the levels of Liquid Culture before freeze-drying. This revealed that C18:1 may mitigate the adverse effects of freeze-drying by elevating the levels of UFAs, particularly MUFAs, thereby safeguarding cells during the process.

Similar variation was also observed in VLCFAs (≥18 carbons). Before freeze-drying, the proportion of VLCFAs accounted for the majority of the total fatty acids on the membrane (77.00% in Liquid Culture). The VLCFAs significantly decreased (*p* < 0.05) to 61.36% after freeze-drying. While in FD-C18:1, it was also significantly higher than that in FD but lower than Liquid Culture. This suggested that the protective effect of C18:1 may include providing the material of VLCFAs (≥18 carbons) to promote the elongation of the fatty acid carbon chain to approach fresh bacteria levels.

The relative expression of genes related to fatty acid synthesis was further analyzed in Fig. 5D. Freeze-drying significantly enhanced (*p* < 0.05) the expression of 3-ketoacyl-ACP reductase *FabG* (LPL9_RS10820) and acyl-*CoA* thioesterase (LPL9_RS10190), from 1.0 (in Liquid Culture) to 1.36 and 4.04, as a response of fatty acid synthesis to freeze-drying stress. While this expression trend was conversed with C18:1 supplementation. The expression of *FabG* and *CoA* in FD-C18:1 decreased to 0.46 and 2.66, indicating C18:1 may provide strong protective effect on membrane fatty acid, thereby alleviating the response to freeze-drying stress of fatty acid synthesis. Moreover, the expression of LPL9_RS10785 was significantly increased after freeze-drying and suppled C18:1, suggesting that L9 responded to freeze-drying stress by increasing the synthesis of cyclopropane fatty acids (C19cyc11), while the addition of C18:1 promoted the synthesis, probably because C18:1 served as sufficient synthetic raw materials. These findings implied that the suppled C18:1 may be converted into long-chain fatty acids on the membrane, reducing the de novo synthesis of fatty acids and increasing the unsaturation of membrane fatty acids.

## Discussion

The present study demonstrated that C18:1 supplementation in the culture medium can significantly improve the fermentation activity of freeze-dried powder of *L. paracasei* L9. Mechanistically, C18:1 regulated fatty acid synthesis-related genes, increasing unsaturated and very-long-chain fatty acid levels in the cell membrane, which enhanced membrane stability and mitigated lyophilization-induced permeability increases. This minimized fermentation-critical enzymes leakage (*e.g.*, *β*-galactosidase), and ultimately preserved intracellular enzymatic activity. This study provides a new strategy for regulating membranes stability and enhancing the freeze-drying tolerance of lactic acid bacteria, and also guiding the industrial production of freeze-drying Directed Vat Set (DVS).

Freeze-drying has been widely reported to impact the survival of *Lacticaseibacillus* species. In our study, we clearly observed that even under the same viable bacterial count, freeze-drying also significantly reduced the fermentation indicators of *L. paracasei* L9. This finding highlights that freeze-drying affects the fermentation ability of live bacterial single cells, a phenomenon that has been largely overlooked. Previous studies have indeed reported similar changes in fermentation-related indicators after freeze-drying while not directly focus on it. For example, Wang *et al*. [31] observed that the acid-producing ability of *L. delbrueckii* subsp. *bulgaricus* was reduced and correlated with increased membrane permeability. This suggested that damage to cell membrane permeability may be a contributing factor to the death and inactivation of LAB during freeze-drying. Basholli *et al*. [32] observed reduced viability and intracellular *β*-glucosidase activity in *B. infantis*, which they associated with membrane damage of integrity and decreased intracellular enzyme retention. These findings provided indirect evidence that fermentation-related functions may also be compromised after lyophilization, highlighting the need to assess both cell viability and fermentation activity. And damage to the membrane stability of LAB during freeze-drying may be a key factor in the loss of fermentation activity observed in freeze-dried strains.

Different strategies have been reported to maintain membrane stability and reduce the loss of viability. For example, trehalose, sodium glutamate, and other components [33] in cryoprotectant elevate the glass transition temperature and penetrate cells to stabilize membranes [34], while optimizing freeze-drying parameters [35], such as pre-freezing and sublimation temperature, could control ice crystal formation and sublimation rate, thereby reducing damage to cell walls and membranes [36]. The protectant employed in this study, adapted with some modifications from a commercial formulation [37], effectively maintained the freeze-drying survival rate of Liquid Culture above 80% and prevented freeze-drying-induced damage from significantly impairing the functionality of the fermentation starter. This formulation provides robust protection through valid components such as the synergistic use of trehalose and sucrose, which helps stabilize the cell structure during freeze-drying. Additionally, the formulation is simple and incorporates commonly used ingredients in the lactic acid bacteria fermentation starter industry, such as skim milk and sucrose, thereby ensuring the industrial relevance and applicability of the research.

However, the optimization of protectants or freeze-drying parameters is merely capable of mitigating the degree of damage to the cell membrane caused by environmental stresses such as low temperature, dehydration, and osmotic pressure during the freeze-drying process. It cannot fundamentally alter the physiological state of the membrane. Since the strains are in a state of low metabolism or dormancy during freeze-drying, protectants function passively, whereas membrane composition and integrity are actively shaped during the culture stage. In contrast to conventional strategies, regulating the metabolism and composition of the strain during the culture process to effectively improve its tolerance during freeze-drying. For example, Hansen *et al*. [38] changed the membrane fatty acid spectrum by culturing *Lactobacillus acidophilus* in MRS with Tween 80, Tween 20, linoleic, or linolenic acid supplemented, to improve storage survival rate. It was found that adding Tween20 could reduce the oxidation of membrane lipids in storage, and the addition of oleic acid increased the robustness of bacteria. E *et al*. [18] also adjusted the buffer system of *L. plantarum* LIP-1 culture medium, and found K^+^ in the buffer salt upregulated the expression of the *luxS* gene in the *LuxS*/autoinducer-2 (AI-2) population sensing system, promoting biofilm formation, thus conferring higher lyophilization survival rate and better storage stability to the strain. These studies support the idea that regulating during culture can influence stress resistance.

The fatty acid composition of membrane phospholipids significantly affects membrane stability. Studies have already demonstrated that altering membrane fatty acid composition through culture conditions can enhance freeze-drying survival rates [39]. For example, UFAs can enhance the mobility of the cell membrane due to their low melting point, thereby reducing permeability and conformational changes in membrane proteins under freeze-drying stress [40]. Chen *et al*. [41] found that supplementation with short-chain fatty acids resulted in the higher population of LAB and lactic acid content during fermentation. This may be attributed to LAB can absorb fatty acids added in the culture medium and convert them into intracellular fatty acids, which may affect cell membrane properties [42]. Liu *et al*. [43] also found that the use of a higher temperature (47°C) or neutral pH (6-7) during fermentation resulted in increased survival of *L. reuteri* I5007 following freeze-drying. An increase in the ratio of UFAs to saturated fatty acids (SFAs) in the bacterial membrane was associated with this higher survival. Additionally, fatty acids interacted with proteins to facilitate the transfer of solutes and regulate the activity of the membrane-bound enzyme Na^+^-K^+^-ATPase [17], which helped maintain normal energy transduction during fermentation metabolism. Therefore, supplementation of culture media with fatty acids may provide a strategy to improve the fermentation activity of strains after freeze-drying by maintaining membrane stability.

In this study, we investigated the differences in membrane fatty acid composition of *L. paracasei* L9 before and after freeze-drying. We found the content of C16:0, C18:1 and C16:1 increased, which is consistent with those of Pei *et al*. [23], who reported that the increment of C16:0 and C18:0 in freeze-dried *Bifidobacterium longum* subsp. *infantis* B8762. And Liu *et al*. [44] also shown that freeze-drying could cause changes in fatty acid composition of *Lactobacillus plantarum* LIP-1. A potential confounding factor is residual fatty acids in skim milk protectant, while the content is very low (less than 0.05%). After washing and centrifugation, the residual content becomes less, and the effect on the analysis of total membrane fatty acids is limited. And the main milk fatty acids that may remain are short-chain fatty acids [45], such as characteristic butyric acid (C4:0) and caproic acid (C6:0). In our study, the fatty acid composition of Liquid Culture and FD were generally similar, especially C8:0. In line with our results, Pei *et al*. [23] also used skim milk as protectant and found the content of C8:0 was similar in liquid-cultured or freeze-dried *B. longum* subsp. *infantis* and C4:0 was even not detected. These results indicated that contribution of skim milk was minor, and the observed changes are predominantly due to the bacteria. And the C18 group significantly enhanced the strain's fermentation activity with using the same protectant as control group (FD), further confirming that the L9 cells, rather than the protectant, played the active role. Thus we chose C16:0, C16:1, and C18:1 added to the culture medium and identified the addition could promote the growth of *L. paracasei* L9 and significantly improved its survival rate after freeze-drying. These findings aligned with the observations reported by Qian [46], who demonstrated that the freeze-drying survival rate of *L. plantarum* N8 was differentially improved when grown in MRS supplemented with Tween 80 (a source of C18:1) or Tween 20 (containing C12:0 and C14:0).

We also found that the addition of C18:1 to the culture medium not only enhanced the viability of freeze-dried L9 cells but also improved their fermentation performance. Importantly, this enhancement was observed under the same initial viable count, indicating a significant improvement in single-cell fermentation capacity and intracellular enzymatic activity (*e.g.*, LDH and *β*-GAL). Previous studies also demonstrated supplementation of culture media with C18:1 fatty acid improved bacterial survival during freeze-drying [47]. For instance, He *et al*.[48] reported that low concentrations of C18:1 increased the viable count and freeze-drying survival rate of *L. plantarum* LIP-1in MRS medium, while Wang *et al*. [49] found that adding 0.001% C18:1 to protectant improved the survival rate of freeze-dried *L. plantarum* to approximately 90%, with enhanced LDH activity and improved membrane integrity. In contrast, our study not only can confirm this protective effect on survival but also demonstrate that fermentation activity—as measured by single-cell fermentation capacity and enzyme activities (LDH, *β*-GAL)—was significantly preserved. And our study further showed that C18:1 enhanced the freeze-drying resistance of L9 by maintaining membrane stability, and consequently improved fermentation activity after freeze-drying, extending the findings of previous studies from membrane protection to functional metabolic preservation. From the aspect of general membrane properties, C18:1 reduced membrane damage and alleviated the increase in cell membrane permeability caused by freeze-drying. The proportion of dead cells with membrane damage was greatly reduced, and the proportion of intact cells also increased. These results confirmed the protective effect of C18:1 on the cell membrane of freeze-dried cells.

In this study, the upregulation of *plsX* and *YfhO* in the FD-C18:1 group suggests their potential involvement in maintaining membrane integrity during freeze-drying. The gene *plsX* encodes an acyl-phosphate synthase that catalyzes the first step of phospholipid biosynthesis. In *Bacillus subtilis* (Gram-positive), *plsX* inactivation reduced biofilm formation ability of the strain [50]. And overexpression of *plsX* significantly increased lipid content in cells by partly overcoming limitation of lipid production [51]. Phospholipids are major structural components of bacterial membranes, forming the lipid bilayer that determines membrane fluidity, barrier function, and resilience under stress.[52] And recent transcriptomic analysis of *Lacticaseibacillus casei* Zhang [53] under VBNC-inducing stress revealed significant *plsX* downregulation, implying that its expression is sensitive to environmental stress and may reflect the cell’s membrane remodeling response. In contrast, the upregulation of *plsX* following C18:1 supplementation in our study may indicate enhanced phospholipid synthesis, contributing to membrane stabilization. Similarly, *YfhO* has been shown to play a role in lipoteichoic acid glycosylation in Gram-positive bacteria such as *B. subtilis* and *Listeria monocytogenes* [54], processes essential for cell envelope structure. While its function has not been confirmed in *L. paracasei*, Tang *et al*. [55] reported increased *YfhO* expression in *Lactobacillus delbrueckii* subsp. *bulgaricus* B61-3 under bovine bone peptone, which was associated with enhanced membrane protein synthesis and fermentation activity. These parallels suggest that *YfhO* may also contribute to cell envelope remodeling and stress resistance in *L. paracasei* L9. Overall, these genes participate in membrane-associated adaptation to freeze-drying stress. Further studies involving targeted gene manipulation will be required to confirm their precise biological functions.

Our study revealed that C18:1 supplementation significantly increased the proportions of UFAs, particularly MUFAs and VLCFAs (≥18C) in the cell membrane. Meanwhile, the expression of *FabG* and *CoA* was downregulated after freeze-drying in the C18:1-supplemented group, approaching those observed in the Liquid Culture group. Since *FabG* encodes 3-oxoacyl-ACP reductase, a key enzyme in the FAS II pathway responsible for elongating fatty acid chains from C4 to C16 or C18 - its downregulation is generally associated with reduced de novo synthesis of long-chain fatty acids. However, we observed a marked increase in VLCFAs from 61.36% in the FD group to 71.88% in FD-C18:1. This contradiction may be explained by the fact that exogenous C18:1 could be directly incorporated into membrane phospholipids, bypassing the endogenous elongation pathway. In addition, the abundance of extracellular UFAs may also exert feedback inhibition on fatty acid biosynthesis regulators such as *FapR* [56], thereby suppressing *FabG* expression. Similar results were also observed by Wang *et al*. [49], who found that exogenous C18:1 can integrate into bacterial membranes and reduce freeze-drying-induced pore formation, thereby enhancing membrane stability. Consistently, our membrane fatty acid profiling showed an increased proportion of C18:1 and other UFAs, suggesting potential integration into the cellular lipid pool and involvement in membrane remodeling. Although the precise mechanism remains to be clarified, this may partially explain the enhanced membrane integrity and fermentation performance observed in the FD-C18:1 group. Moreover, the downregulation of *FabG* and *CoA* may reduce the metabolic burden associated with fatty acid elongation, conserving ATP and NADPH for other protective processes such as trehalose biosynthesis [57] and molecular chaperone production[58], which collectively enhance freeze-drying tolerance. This consistency in expression changes further supports the notion that FAS II pathway activity was globally suppressed following C18:1 supplementation, likely reflecting a reduced demand for acetyl-*CoA* as a substrate. In addition, the relative expression of LPL9_RS10785 in FD-C18:1 was 1.5 times that of FD, which encodes cyclopropane-fatty-acyl-phospholipid synthase family protein in L9. It is consistent with the findings of Qiao *et al*. [9], who reported low concentrations of C18:1 promoted cyclopropane fatty acid synthesis and increase membrane unsaturation through cyclic structures, improving lateral mobility. Elevated UFA levels have also been associated with increased membrane viscosity and thickness, which help reduce permeability [19] and enhance membrane resistance during lyophilization.

This study revealed that C18:1 supplementation mitigated freeze-drying-induced damage to membrane integrity and permeability by enhancing membrane stability. The reduced leakage of intracellular enzymes (*e.g.*, *β*-galactosidase) collectively contributed to improved activity of fermentation-related enzymes. These effects not only elevated the survival rate of *L. paracasei* L9 after lyophilization but also significantly enhanced its post-lyophilization fermentation performance. The underlying mechanism involved C18:1-mediated regulation of membrane associated genes (*YfhO* and *FabG*), which increased the proportion of monounsaturated fatty acids (MUFAs) and long-chain fatty acids, thereby stabilizing membrane structure and function. To further illustrate the industrial relevance of this enhancement, we compared the recovery levels of key fermentation indicators in this study with those reported in our previous work [59] using a different commercial protectant system [60, 61]. In that earlier study, the freeze-dried *L. paracasei* L9 preserved with industrial protectants recovered 69% of *β*-galactosidase activity, 55% of LDH activity, and 91% of titratable acidity, relative to its liquid-cultured counterpart. In contrast, the FD-C18:1 group in the present study achieved higher recovery levels of 86.5%, 99.8%, and 99.6%, respectively, for the same parameters. While the protectant formulations differed between the two studies, this internal normalization allows for a valid performance comparison. These findings suggest that C18:1 supplementation may provide additional benefits to conventional protectants by further enhancing post-lyophilization fermentation activity, thereby supporting its potential utility as a functional additive in industrial starter formulations.

In conclusion, supplementing C18:1 in the culture medium represents an effective and industrially applicable approach to enhance the freeze-drying tolerance of *L. paracasei* L9. By regulating membrane-associated gene expression (*YfhO* and *FabG*) and optimizing membrane fatty acid composition (increased MUFAs and VLCFAs), C18:1 strengthened membrane stability, reduces lyophilization damage, and ultimately improves both intracellular enzyme activity and fermentation capacity per viable cell. These results provided the basis for industrial application of *L. paracasei* L9 and production of freeze-dried powder products.

## Supplemental Materials

Supplementary data for this paper are available on-line only at http://jmb.or.kr.



## Figures and Tables

**Table 1:** Membrane fatty acid composition of *Lacticaseibacillus paracasei* L9 before and after freeze-drying.

Fatty acid(s)	Percentage of total (%)	
	Liquid Culture	FD
C6:0	2.05 ± 0.09	1.44 ± 0.07
C12:0	2.73 ± 0.30	3.75 ± 0.34
C14:0	2.46 ± 0.33	2.43 ± 0.39
C16:0	27.80 ± 0.80	30.98 ± 0.77*
C16:1	1.31 ± 0.06	4.69 ± 0.09*
C18:0	10.42 ± 0.61	12.58 ± 0.50*
C18:1	43.11 ± 2.16	36.65 ± 0.49*
C18:2	8.20 ± 0.20	5.22 ± 0.09*
C18:3	0.82 ± 0.24	1.42 ± 0.07
C20:0	1.10 ± 0.05	0.84 ± 0.07

Data were shown as mean ± standard deviation of independent triplicate. *Denotes statistical difference (*p* < 0.05), based on the *t*-tests.
