# Supplementary material for: Supplement C18:1 in Culture Medium to Improve Survival Rate and Fermentation Activity of Lyophilization of Lacticaseibacillus paracasei L9
Source: J Microbiol Biotechnol. 2025 Aug 28;35:e2505007. doi: 10.4014/jmb.2505.05007 (PMC12409430; doi:10.4014/jmb.2505.05007)
Supplement: Supplementary file 1 [file jmb-35-e2505007-supple.pdf]

Supplementary Fig. 1 Cell membrane damage of different subgroups of *Lacticaseibacillus paracasei* L9 after freeze-drying

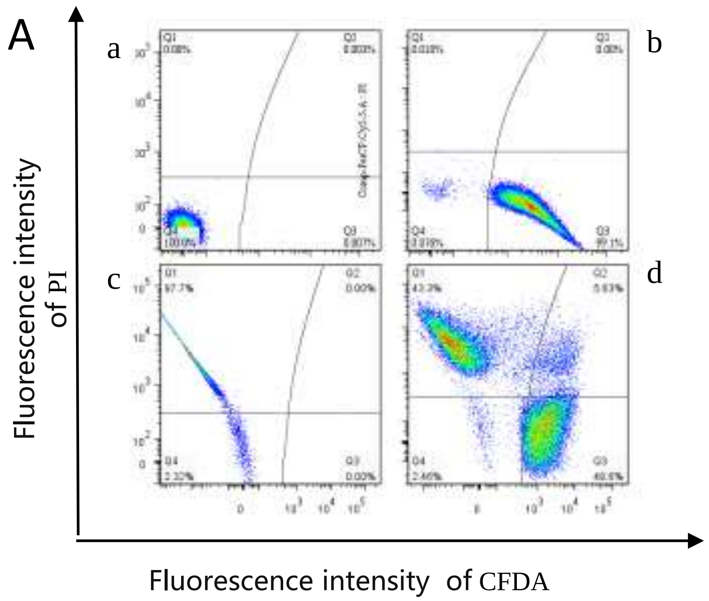

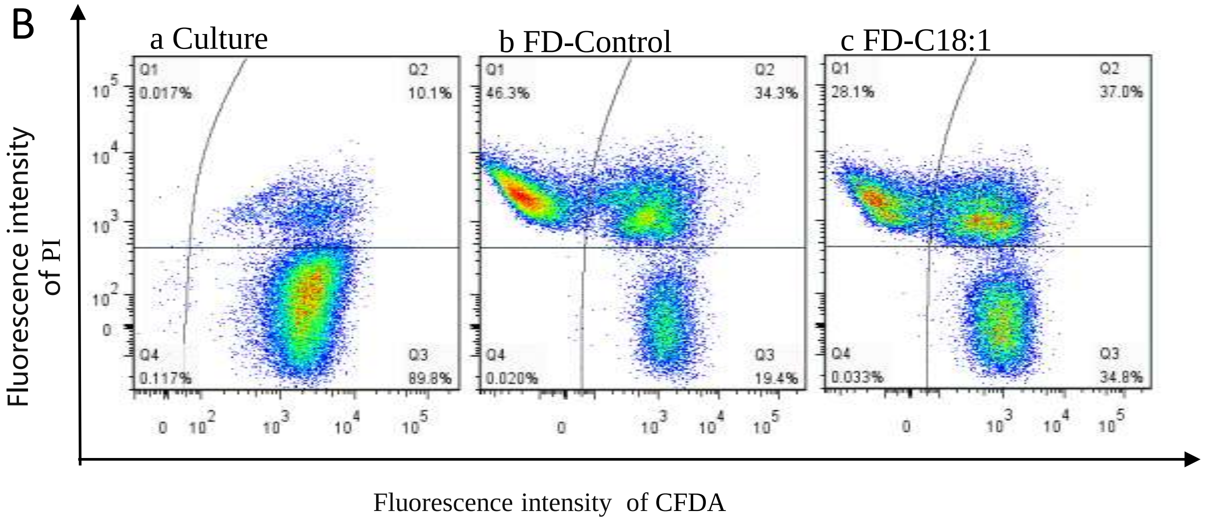

Note: SF.1A was used to adjust parameters of FCM, and the cell membrane damage of Culture(SF.1Ba), FD-Control(SF.1Bb) and FD-C18:1(SF.1Bc) are shown in SF.1B.

Q1 indicates the dead subgroup; Q2 indicates the damaged subgroup; Q3 indicates the intact subgroup and Q4 indicates the negative control in SF.1.

FD- indicates groups with freeze-drying treatment. USFA: unsaturated fatty acid; MUSFA: monounsaturated fatty acid; LCFA: long chain fatty acid; SCFA: short chain fatty acid.

[a-c] Different letters indicate significant differences (p <0.05) between L9 with different treatments, based on the Tukey test.

Error bars represent standard deviation obtained using independent triplicates.

Supplementary Table. 1 Primers used in the RT-PCR

Gene	Primer Names and Primer Sequences (5’-3’)
16s rRNA	16sF: AAAATCGCCACACCGTGC
	16sR: GCAAACAGAGCTCAGCCAAG
LPL9_RS08900	8900F: TATGCTTTGAGTGGCTGGCT
	8900R: TCAATAATGAGCGGCAGCAG
LPL9_RS05860	5860F: TCAGACTGATGGTGAAGGTCAT
	5860R: CGCGATTTAAAAAGCATCGAAC
LPL9_RS10190	10190F: ACCAGTGCATCCCCCATTTT
	10190R: AGTTCAATTOCCOCACAACO
LPL9_RS10820	10820F: GCCCGCTTTACTAGCTGCAT
	10820R: GGCATGCACTGGCGTCATTA
LPL9_RS08660	8660F: CCGTCCGTCACCACAACAT
	8660R: CGCGGCGTTGAAAATCCAC
LPL9_RS10785	10875F: ACTGTTCCTTOCTCAGOGTG
	10875R: GGCTGGGGTACGTTGATGTT

Supplementary Table. 2 Membrane fatty acid composition of freeze-dried *Lacticaseibacillus paracasei* L9 suppled with C18:1

Fatty acid(s)	Percentage of total(%)		
	Culture	Freeze-dried Control	Freeze-dried C18:1
C6:0	0.138±0.003	0.135±0.005	0.152±0.015
C8:0	——	0.694±0.023	0.63±0.011
C10:0	——	2.082±0.102[a]	1.974±0.084[b]
C12:0	0.416±0.013[b]	3.683±0.054[a]	3.373±0.114[a]
C13:0	0.151±0.029[b]	0.739±0.023[a]	0.752±0.083[a]
C14:0	9.044±0.123[b]	12.057±0.117[a]	7.252±0.185[c]
C14:1	0.360±0.01	0.358±0.01	0.359±0.009
C15:0	0.242±0.006[b]	0.745±0.015[a]	0.716±0.027[a]
C16:0	8.687±0.122[c]	15.084±0.076[a]	11.09±0.064[b]
C16:1	1.154±0.013[b]	2.183±0.085[a]	0.845±0.015[c]
C17:0	0.404±0.015	0.549±0.006	0.572±0.032
C17:1	0.405±0.011	0.329±0.012	0.402±0.036
C18:0	8.502±0.41[b]	9.803±0.046[a]	9.25±0.634[ab]
C18:1n9t	5.238±0.317	5.381±0.123	5.561±0.593
C18:1n9c	56.548±0.793[a]	39.394±0.313[c]	49.342±2.644[b]
C18:2n6c	6.771±0.382[a]	4.643±0.142[c]	5.693±0.672[b]
C18:3n3	——	0.391±0.027	0.445±0.043
C20:0	0.476±0.017	0.301±0.002	0.315±0.031
C20:1n9	1.046±0.232	0.447±0.012	0.317±0.038
C22:0	——	0.23±0.009[b]	0.286±0.032[a]
C20:5n3	——	0.237±0[b]	0.314±0.04[a]
C24:0	0.418±0.019	0.537±0.312	0.361±0.042
UFA	71.521±0.342[a]	53.363±0.328[c]	63.277±1.253[b]
MUFA	64.75±0.644[a]	48.091±0.163[c]	56.826±1.986[b]
PUFA	6.771±0.382[a]	5.271±0.168[b]	6.452±0.755[a]
≥18C	76.999±0.226[a]	61.363±0.093[c]	71.884±0.579[b]
＜18C	23.001±0.226[c]	38.637±0.093[a]	28.116±0.579[b]

Note : Data were shown as mean ± standard deviation of three independent experiments performed in triplicate.

[a-c] Different letters indicate significant differences (p <0.05) between L9 with different treatments, based on the Tukey test.

—Denotes the content is lower than the lower limit of detection.
